# Role of adventitious roots in water relations of tamarack (*Larix laricina*) seedlings exposed to flooding

**DOI:** 10.1186/1471-2229-12-99

**Published:** 2012-06-27

**Authors:** Mónica Calvo-Polanco, Jorge Señorans, Janusz J Zwiazek

**Affiliations:** 1 Department of Renewable Resources, University of Alberta, 442 Earth Sciences Bldg, Edmonton, AB T6G 2E3, Canada

## Abstract

**Background:**

Flooding reduces supply of oxygen to the roots affecting plant water uptake. Some flooding-tolerant tree species including tamarack (*Larix laricina* (Du Roi) K. Koch) produce adventitious roots in response to flooding. These roots were reported to have higher hydraulic conductivity under flooding conditions compared with non-adventitious roots. In the present study, we examined structural and functional modifications in adventitious roots of tamarack seedlings to explain their flooding tolerance.

**Results:**

Seedlings were subjected to the flooding treatment for six months, which resulted in an almost complete disintegration of the existing root system and its replacement with adventitious roots. We compared gas exchange parameters and water relations of flooded plants with the plants growing in well-drained soil and examined the root structures and root water transport properties. Although flooded seedlings had lower needle chlorophyll concentrations, their stomatal conductance, net photosynthesis rates and shoot water potentials were similar to non-flooded plants, indicative of flooding tolerance. Flooded adventitious roots had higher activation energy and a higher ratio of apoplastic to cell-to-cell water flow compared with non-flooded control roots as determined with the 1-hydroxypirene 3,6,8-trisulfonic acid apoplastic tracer dye. The adventitious roots in flooded plants also exhibited retarded xylem and endodermal development and accumulated numerous starch grains in the cortex. Microscopic examination of root sections treated with the PIP1 and PIP2 antibodies revealed high immunoreactivity in the cortex of non-flooded roots, as compared with flooded roots.

**Conclusions:**

Structural modifications of adventitious roots suggest increased contribution of apoplastic bypass to water flow. The reduced dependence of roots on the hypoxia-sensitive aquaporin-mediated water transport is likely among the main mechanisms allowing tamarack seedlings to maintain water balance and gas exchange under flooding conditions.

## Background

Flooding creates hypoxic conditions around the roots affecting a number of physiological processes in plants including gas exchange, carbohydrate metabolism and water relations [1-3]. Some woody plants that are adapted to flooding conditions develop hypertrophic lenticels and/or root aerenchyma to increase aeration [4,5]. In other species, including tamarack (*Larix laricina* (Du Roi) K. Koch), flooding triggers the development of adventitious roots, which help the trees tolerate seasonal changes in water levels [6]. However, the mechanisms through which adventitious roots contribute to flooding tolerance of the tree and those that enable the roots to survive hypoxic conditions remain unclear. Adventitious roots in tamarack are often present below the water level for extended periods of time [7,8] and, therefore, they must be adapted to low oxygen conditions.

Stomatal closure and wilting are among the initial symptoms of oxygen deficiency in the root zone [1,9] due to the reduced ability of the root system to conduct water [10-12]. The maintenance of fine balance between the water loss and water uptake requires adjustments in tissue hydraulic conductivity. Most of the dynamic regulation of root hydraulic conductivity has been attributed to the transmembrane water flow regulated by the aquaporins. The reduction of root hydraulic conductivity in hypoxic plants has been linked to the inhibition of aquaporin-mediated water transport through root metabolic changes [12,13] and low cytoplasmic pH [14], and could be partly alleviated by the treatment of plants with ethylene [11,15]. In flooded tamarack seedlings, the emergence of adventitious roots coincided with an increase in hydraulic conductance of the root system [16]. Adventitious roots in tamarack were also reported to have higher hydraulic conductivity (conductance expressed on the root volume basis) under flooding conditions compared with non-adventitious roots of the same tree [16] suggesting that adventitious roots may posses functional modifications which make them less sensitive to flooding.

Since aquaporins are sensitive to hypoxia [12,13], the adaptations of adventitious roots to flooding likely include modifications that are aimed at making the roots less dependent on the transmembrane water transport. An increase in apoplastic water pathway could reduce the dependence of root water transport on aquaporins, but it could also compromise the benefits of selective permeability of the transmembrane pathway. In the present study, we examined the hypothesis that the adventitious roots produced in tamarack in response to flooding are able to maintain high hydraulic conductivity by developing structural and functional modifications that increase the apoplastic bypass. In turn, these features enable flooded plants to maintain stomatal conductance and photosynthesis to sustain carbohydrate supply to the roots. We subjected seedlings to six months of flooding, which resulted in the replacement of the existing root system with newly-produced adventitious roots. We then compared the gas exchange and water relations of flooded plants with the plants growing in a well-drained soil and examined the differences in root structure and relative contributions of apoplastic and cell-to-cell pathways in flooded and non-flooded plants.

## Results

### Dry weights, gas exchange, water potentials and leaf chlorophyll concentrations

Flooded seedlings showed a transient inhibition in shoot growth and photosynthetic rates which was accompanied by an increase in leaf necrosis during the first month of the flooding treatment (data not shown), but the seedlings recovered in the following months. After six months of flooding, there were no statistically significant differences in root, shoot and total dry weights between flooded and non-flooded plants (Table 1).

**Table 1:** **Seedlings dry weights, needle chlorophyll content (NC), net photosynthesis (NP), transpiration (E), water potential (WP), whole root system hydraulic conductance (K**_**TOT**_**) and hydraulic conductivity (L**_**TOT**_**) in flooded adventitious roots and non-flooded control roots of tamarack seedlings**

	**Non-flooded**	**Flooded**
Root DW (g)	13.8 ± 0.5	11.8 ± 1.2
Shoot DW (g)	26.5 ± 1.7	25.7 ± 3.5
Total DW (g)	39.5 ± 2.3	38.3 ± 4.7
Shoot:Root Ratio	1.9 ± 0.1	2.3 ± 0.3
NC (mg g^-1^ DW)	14.7 ± 1.0^a^	9.4 ± 0.7^b^
NP (μmol CO_2_ m^-2^ s^-1^)	5.6 ± 1.2	6.4 ± 1.5
E (mmol H_2_O m^-2^ s^-1^)	0.5 ± 0.1	0.6 ± 0.1
WP (MPa)	−0.78 ± 0.04	−0.81 ± 0.05
K_TOT_ (kg MPa^-1^ s^-1^ × 10^-5^)	1.2 ± 0.2	1.2 ± 0.4
L_TOT_ (kg MPa^-1^ cm^-3^ s^-1^ × 10^-7^)	2.0 ± 0.3	1.6 ± 0.1

Different letters within the same row indicate significant differences between flooded and non-flooded plants (*t*-test, *p* = 0.05). Values are means ± SE (n = 6 for K_TOT_ and L_TOT_ and n = 7 for the rest of the measurements).

Needle chlorophyll concentrations were significantly lower in flooded compared with non-flooded seedlings after six months of treatment (Table 1). However, there were no significant differences in net photosynthesis, transpiration rates and shoot water potentials between the two groups of plants (Table 1).

### Root hydraulic properties

**Figure 1:**
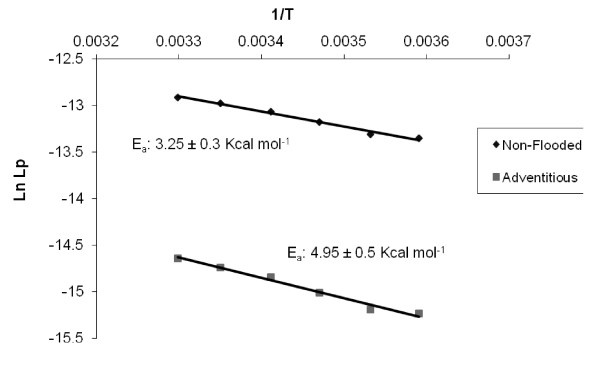
**Arrhenius plots in individual roots of non-flooded tamarack seedlings and in adventitious roots of flooded tamarack seedlings.** Means (n = 6) ± SE are shown.

There were no significant differences in the hydraulic conductivity (L_TOT_) of the whole root systems between non-flooded and flooded seedlings when measured after six months of flooding (Table 1). However, at all measured temperatures ranging from 10 °C to 30 °C, the hydraulic conductivity (L_IND_) of individual roots (20 – 30 cm long) was several-fold higher in non-flooded seedlings compared with adventitious roots of flooded plants (Figure 1).

Activation energy (E_a_, kcal mol^-1^) for L_IND_ was higher in adventitious roots of flooded plants (4.95 ± 0.5) compared with control non-flooded roots (3.25 ± 0.3) (Figure 1). Similarly, the PTS_3_ concentration of xylem exudates (%) was almost two-fold higher in flooded adventitious roots (7.46 ± 1.1 ×10^-3^) compared with the roots of non-flooded seedlings (4.12 ± 0.8 ×10^-3^).

### Root morphology and structure

**Figure 2:**
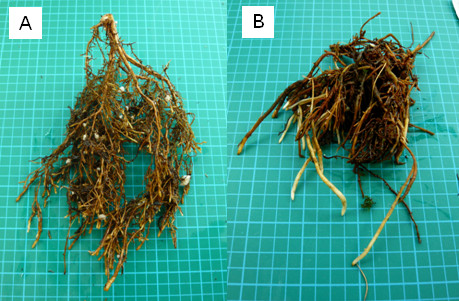
Roots in non-flooded tamarack seedlings (A) and adventitious roots in flooded plants (B) after six months of the flooding treatment.

Most of the existing roots disintegrated in plants during the first several months of flooding except for the older woody parts of the roots which comprised about one-third of the whole root system in flooded plants. The adventitious roots were distributed close to the soil surface. Control roots consisted of numerous relatively short, mostly woody and branched roots with relatively short white tips (2–4 cm) (Figure 2A). The root system in flooded plants consisted of fewer but longer (up to 30-cm long) adventitious roots (Figure 2B), largely unsuberized and with no secondary tissues present for as far as 10–15 cm above the root apex.

**Figure 3:**
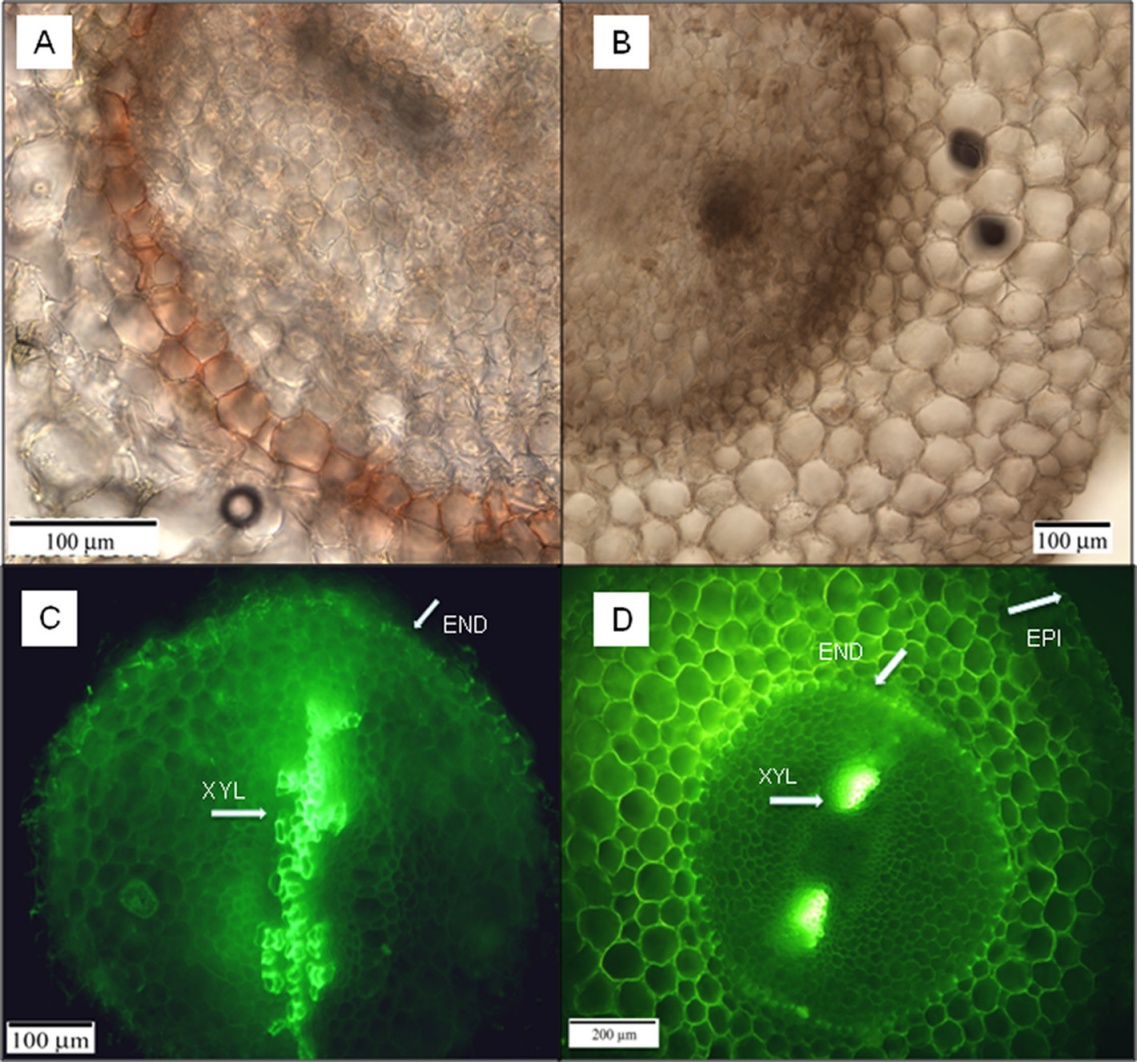
**Cross sections of non-adventitious non-flooded roots (A,C) and flooded adventitious roots (B,D) showing reduced size of vascular bundles and poorly-developed endodermis in the adventitious root.** The sections were taken at 0–0.2 cm from the root tip (**A,B**) and 1 cm from the root tip (**C,D**). The root sections were stained with Sudan IV (**A,B**) and berberine (**C,D**). The sections for Sudan IV were mounted on slides and examined under the light microscope. The sections for berberine were examined under the fluorescence microscope (Leica DMRXA Upright Microscope) with a green light filter I3 at 450–490 nm excitation and 510 nm emission. Epidermis (EP), Endodermis (EN) and xylem (XYL) structures are indicated with arrows.

There were several major structural differences in the zone of the primary tissues between non-flooded roots and adventitious roots. The non-flooded roots contained a well-developed endodermis with suberized cell walls (Figure 3A) and well-developed xylem (Figure 3C) that could be observed close to the root tips. Adventitious roots of flooded plants had poorly-developed endodermis (Figure 3B) and few, small-diameter, tracheids throughout the examined length (up to 5 cm from the root tip) of the root (Figure 3D).

**Figure 4:**
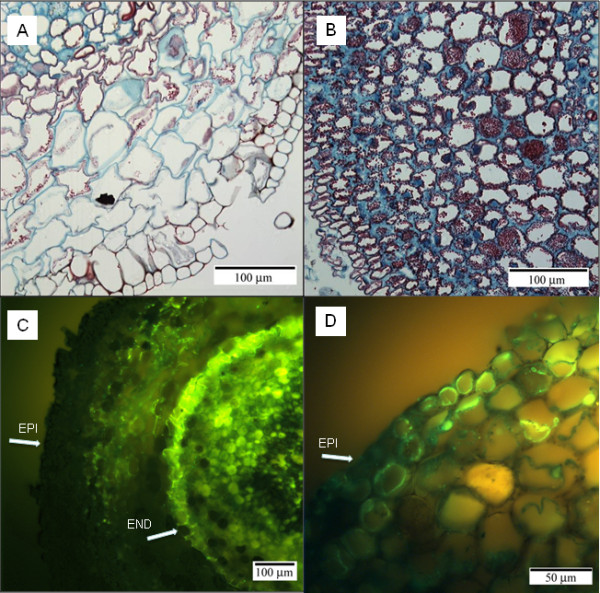
**Cross sections of non-adventitious roots from control, non-flooded-plants (A,C) adventitious roots from flooded seedlings (B,D).** The Safranin O and Fast Green FCF sections show starch accumulation in the cortical cells at 0.5 cm from the root tip (**A,B**). These sections were mounted on slides and examined under the light microscope. Immunolocalication of PIP1aquaporins (**C,D**) was carried out with sections taken at 0.5 cm from the root tip. The sections were examined under the fluorescence microscope (Leica DMRXA Upright Microscope) with a green light filter I3 at 450–490 nm excitation and 510 nm emission. Epidermis (EP), Endodermis (EN) and xylem (XYL) structures are indicated with arrows.

There was relatively little starch present in the root cells of non-flooded plants (Figure 4A). In contrast, the cortex of flooded adventitious roots was filled with starch grains (Figure 4B).

### Inmunolocalization of root aquaporins

PIP1 and PIP2 antibodies showed similar immunostaining patterns in roots and, therefore, only the PIP1 antiserum is shown. The Western blot analysis showed an immunoreactive band in maize and tamarack root proteins separated by SDS PAGE ( Additional file 1). In both species, the band of a molecular weight corresponding to an aquaporin monomer of approximately 30 kD was visible and another, faint, band of approximately 60 kDa corresponding to the aquaporin dimer. The most intense immunofluorescence in the adventitious roots of flooded plants that were incubated with PIP1 and PIP2 antibodies was present in the outer cortical cells and it was less intense in other parts of the root compared with the non-flooded roots (Figure 4D). In non-flooded roots, PIP1 and PIP2 aquaporins were abundant in the root cortex and concentrated especially in its central part away from the epidermis and endodermis (Figure 4C). The stele in adventitious roots and in non-flooded roots incubated with PIP1 and PIP2 also showed relatively intense immunofluorescence.

## Discussion

After a transient inhibition of shoot growth, photosynthesis and the increase in needle necrosis, the recovery and resumption of shoot growth in flooded tamarack coincided with the initiation of adventitious roots. Since the existing younger roots disintegrated over time, there were only old woody roots and new adventitious roots that were present in seedlings at the end of the flooding treatment. Even though needle chlorophyll concentrations were reduced by the flooding treatment, transpiration rates, net photosynthesis, and shoot water potentials were similar in flooded and non-flooded plants. These results suggest that, despite some hypoxia-induced toxicity symptoms, adventitious roots maintained adequate water supply to flooded seedlings. Flooding commonly triggers reductions in leaf chlorophyll concentrations in plants [4,17]. The lack of an effect of reduced needle chlorophyll concentrations on net photosynthesis in flooded tamarack suggests that stomatal factors are likely to override photosynthetis responses to flooding under the study conditions.

The initial responses of plants to flooding include an inhibition of gas exchange [18,19]. However, transpiration and photosynthesis have been often reported to recover in flooding-tolerant plants [1,20] suggesting that the tolerant plants can restore their water balance over time. The recovery of root hydraulic conductance in flooded tamarack seedlings coincided with the emergence of adventitious roots suggesting that adventitious roots were more flooding-tolerant compared with non-adventitious roots [16]. This recovery is usually related with the production of ethylene within the roots that will induce an increase on root water transport [11] and hence an increase on the phosphorylation of aquaporins. Hypoxia, which is the main consequence of flooding, inhibits root water uptake through its effect on the aquaporin-mediated water transport due to low cytosolic pH and inhibition of respiration [11,14,21]. Therefore, adaptations of the root system to flooding may be expected to include the structural and functional modifications which decrease the dependence of the root system on the aquaporin-mediated water transport.

In the present study, a large part of the root system in flooded tamarack seedlings consisted of long, non-woody, adventitious roots with a large unsuberized absorption surface. As part of the adaptations to flooding, the adventitious roots contained greatly reduced vascular bundles when examined as far as 3 cm from the root apex. Also, their endodermal layer was poorly developed and often not apparent when examined at the same distance from the root apex as the roots of non-flooded plants. The fact that the cortex of adventitious roots contained abundant starch grains suggests that they were the sink for carbohydrates. Starch is a primary energy storage compound and its allocation pattern and translocation may be critical for growth and hypoxia tolerance [22]. It has been suggested that plant survival in wetland habitats may depend on root carbohydrate reserves [23] and starch abundance is considered to be among the principal characteristics of flooding-tolerant tree species [24,25]. High accumulation of starch in the rooting regions has been also associated with adventitious root formation [26,27]. It is plausible that large amounts of starch are needed to supply adventitious roots with sufficient energy required to support their growth and basic metabolic functions under hypoxic conditions. However, a possible effect of starch accumulation on matric potential of the root cortex and its consequences for root water relations also deserve further attention.

Both K_TOT_ and L_TOT_ of the whole root systems were similar in flooded and non-flooded plants (Study 1). When measured in individual roots, K_IND_ and L_IND_ were several-fold lower in flooded adventitious roots compared with non-flooded control roots (Study 2). Therefore, there were likely water entry points through the parts of the old, partly disintegrated root system. Regardless of the differences in the hydraulic conductance of flooded and non-flooded plants, growth, gas exchange, and shoot water potentials suggest that water transport was not the limiting factor to the flooded plants with established adventitious roots. This was likely facilitated by the greater water absorption area of adventitious roots and structural modifications increasing the apoplastic bypass.

The results of PTS_3_ and immunostaining with the PIP1 and PIP2 antibodies support the notion of the reduced role of cell-to-cell water transport in adventitious flooded roots. The concentration of PTS_3_ in the xylem sap expressed from the individual flooded adventitious roots was almost two-fold higher compared with control non-flooded roots. PTS_3_ is a water-soluble fluorescent, non-toxic dye that does not cross cell membranes or adhere to cell walls [28,29]. Although the exact concentrations of apoplastic flow calculated from the fluorescent tracer dye concentrations are not precise estimates of the actual apoplastic flow rates, they have been used to estimate relative changes in water flow pathways [10,30-32]. Increases in PTS_3_ concentrations in the xylem sap have been also correlated with the inhibition of aquaporin-mediated transport in plants exposed to drought [30,31], mercury [31], and metabolic inhibitors [10,33].

Higher activation energy for water flow in flooded adventitious roots (4.95 kcal mol^-1^) compared with non-flooded control roots (3.25 kcal mol^-1^) suggests that water transport in flooded adventitious roots is more sensitive to temperature compared with the non-flooded roots. For the transmembrane transport, E_a_ increases with increasing restriction of water movement through aquaporins [34,35] and the overexpression of PIP1 and PIP2 aquaporins in *Arabidopsis* abolishes temperature sensitivity of root cell hydraulic conductivity over the range of 10 to 25 °C (unpublished results). However, at the whole root level, hydraulic responses to temperature are confounded by the effects of apoplastic pathway [36,37].

The immunolocalization of PIP aquaporins showed that in addition to the overall reduced intensity, immunofluorescence was concentrated in the outer parts of the cortex and epidermis of adventitious flooded roots. This suggests their relatively greater role in the root surface water uptake as demonstrated for PIP2;5 in maize [38]. A more uniform distribution and higher intensity of immunostaining throughout the root cortex of non-adventitious non-flooded roots combined with the presence of larger vascular bundles and well-developed endodermis suggests a greater functional importance of radial water transport in this root zone.

The ability to produce adventitious roots offers an opportunity to tamarack to develop the features that allow trees to survive flooding conditions. It remains to be determined whether the modifications in water transport properties constitute a general flooding tolerance mechanism or are unique to this tree species.

## Conclusions

The study has demonstrated that after six months of flooding, tamarack seedlings had similar shoot water potentials, transpiration rates and net photosynthesis to non-flooded plants. Flooded seedlings produced adventitious roots which accumulated numerous starch grains in the cortex and developed structural modifications which likely contributed to their reduced dependence on the aquaporin-mediated water transport under hypoxic conditions. The reduced dependence of roots on the hypoxia-sensitive aquaporin-mediated water transport is likely among the main mechanisms allowing tamarack seedlings to maintain water balance and gas exchange under flooding conditions.

## Methods

### Plant material and growth conditions

Six-month-old tamarack seedlings were obtained from the Bonnyville Forest Nursery Inc., AB and stored frozen over winter. In spring, the seedlings were transferred into 4.5 L pots (Nursery Supplies, Tustin, CA) filled with commercial soil mixture (Sunshine LA4 Mix, Sun Grow Horticulture Canada Ltd, Spruce Grove, AB, Canada). The pots were placed in a greenhouse that was maintained at 22/18 °C day/night temperature, 18-h photoperiod, 65 ± 10% relative humidity. Natural light was supplemented with 400-W high pressure sodium lamps giving a minimum photosynthetic photon flux density (PPFD) of 350 μmol m^-2^ s^-1^ (Lumalux, GTE Sylvania, Drummondville, PQ, Canada). The seedlings were fertilized once a week with the 20:20:20 (N:P:K) commercial fertilizer applied at 0.25 g L^-1^.

After one month, a group of 20 seedlings was subjected to flooding for six months. The flooding treatment was applied by immersing the pots in mineral solution up to about 5 cm below the top edge. The mineral solution was changed once a week. The initial oxygen concentration of the solution after each weekly change was about 8 mg L^-1^, and declined over the course of the week to approximately 2–3 mg L^-1^.

A second group of 20 seedlings served as non-flooded control. The pots with control plants were placed on a greenhouse bench next to the flooded plants and the plants were regularly watered and fertilized as above. The experiment was repeated three times in three different years at the same times during the growing season (Study 1, 2, and 3).

### Dry weights, gas exchange and leaf chlorophyll concentrations

Plant dry weights, gas exchange and leaf chlorophyll concentrations were measured after six months of flooding in Study 1. For dry weight determinations, roots were separated from shoots in six seedlings per treatment (n = 6) and their dry weights obtained after drying at 65 °C for 72 h.

Net photosynthesis (NP) and transpiration (E) rates were measured in the greenhouse between 10:00 and 11:30 using the uppermost branches of six seedlings (n = 6) per treatment after six months of the flooding treatment. At that time, the upper branches of flooded plants did not shown signs of needle chlorosis or necrosis, which were already apparent in the lower branches. For the gas exchange measurements, an infrared gas analyzer (LCA-4, Analytical Development Company Ltd., Hertfordshire, UK) was used with an auxiliary light source (1000 μmol m^−2^ s^−1^ PPFD). Needle surface areas were determined with the Sigma Scan 5.0 software following scanning (Jandel Scientific, San Rafael, CA).

Shoot water potential was measured with a Scholander pressure chamber in the upper lateral branches of the trees. The branches were attached from the stems into the pressure chamber and compressed air was applied. The water potential was determined as the pressure needed for the first drop of water to come out from the branch stem.

Needle chlorophyll concentrations were determined in six seedlings per treatment (n = 6) in the same needles as those used for the gas exchange measurements. The needles were freeze-dried and grinded. For chlorophyll determinations, 20 mg DW of tissue was extracted with 8 ml methanol. The methanol extracts were analyzed spectrophotometrically (Ultrospec III, Pharmacia LKB, Uppsala, Sweden) and the MacKinney’s equation [39] was used for the calculations of needle chlorophyll concentrations.

### Root hydraulic conductance (K_TOT,_ K_IND_), conductivity (L_TOT,_ L_IND_) and xylem exudate concentrations of 1-hydroxypirene-3,6,8 – trisulfonic acid (PTS_3_)

Whole root system hydraulic conductance (K_TOT_) and conductivity (L_TOT_) were determined after six months of flooding treatment in six flooded and six non-flooded plants (n = 6) in Study 1. The plants were measured at room temperature from 10:00 to 16:00. The shoots of seedlings were excised about 1.5 cm above the root collar without removing the roots from the soil. The roots were attached to the high pressure flow meter (HPFM, Dynamax Inc., Houston, TX) for K_TOT_ measurements. Each root system was gradually pressurized to 0.5 MPa [40]. After the measurements, root volumes were calculated using the volume displacement method [33] and L_TOT_ was calculated.

For Study 2, root hydraulic conductance measurements were carried out with a HPFM on sets of individual roots (K_IND_) cut at the stem level (Figure 2) of six non-flooded plants and on six adventitious roots from flooded seedlings (n = 6). For the measurements, the roots were placed in a water bath at 10 °C and their K_IND_ was determined at increasing temperatures from 10, 15, 20, 25, to 30 °C. The roots were held for 10 minutes after each temperature change before the measurement. Root hydraulic conductivity (L_IND_) was calculated based on the root volume calculated by the volume displacement method as explained above.

The activation energy (E_a_, kcal mol^-1^) for root water transport was calculated from the Arrhenius plots of the natural logarithm of L_IND_ values against the inverse of absolute temperatures [41].

The apoplastic tracer dye 1-hydroxypirene-3,6,8 – trisulfonic acid (PTS_3_) was used to examine relative changes in the apoplastic to cell-to-cell water flow ratio in the roots. PTS_3_ is a fluorescent dye that is not transported across the cell membranes and which have been often used to estimate changes in ratio of apoplastic measurements of cell-to-cell water transport in roots. For the measurements, we used the same sets of roots as in the measurements of L_IND_. The roots were placed in a Scholander pressure chamber in an aqueous solution of 0.02% (w:v) PTS_3,_ and pressurized at 0.3 MPa for 10 min [31]. The exudates were collected and PTS_3_ concentrations were determined using a Sequoia-Turner 450 fluorometer (Apple Scientific, Chesterland, OH, USA) with 405 nm excitation and 515 nm emission spectra [28].

### Root anatomy and root starch content

Root tips of eight control-non flooded seedlings and eight distal root segments of adventitious and non-adventitious flooded seedlings were randomly selected after 6 months of flooding treatment (between 0.5- and 1-cm long in Study 1 and up to 5-cm long in Study 3). In Study 1, the root segments were fixed with FAA (70% ethanol, glacial acetic acid and formalin) overnight followed by 4% paraformaldehyde and 0.5% glutaraldehyde for 6 h. After fixation, the root segments were dehydrated in an ethanol series, placed in toluene and embedded in paraffin. Serial cross sections (6-8-μm thick) of paraffin-embedded roots were prepared with a microtome. The paraffin sections were cleared with toluene and 95% ethanol and stained with 0.1% Safranin O (in 95% ethanol (w:v) for 1 h follow by 0.1% Fast Green FCF in 95% ethanol (w:v) for 1 min. The sections were mounted on slides and examined under the light microscope.

The development of xylem and endodermal tissues were examined in Study 3. Root segments were obtained at different distances from the fresh root tips (0, 0.5, 1, 1.5, 2, and 3 cm). Fresh, free-hand sections were prepared with a razor blade and stained with Sudan IV or Berberine [42]. The sections were examined with the light and fluorescent microscope (Leica DMRXA Upright Microscope) using a green light filter I3 at 450–490 nm excitation and 510 nm emission.

Starch content and distribution was examined in fresh, free-hand sections taken up to 1 cm from the root tip of non-flooded roots and adventitious roots from flooded plants. The sections were stained with IKI and viewed under the light microscope.

### Inmunolocalization of root aquaporins

For in situ immunolocalization of aquaporins, fresh free-hand sections were prepared with a razor blade from the roots of six non-flooded and flooded seedlings. The sections were taken from 0.5 to 1 cm from the root tip. They were incubated with the antibodies raised against PIP1 and PIP2 aquaporins [38]. The anti-PIP1 antibody (R-4445) was raised against an amino peptide from ZmPIP1;5 and recognizes all maize PIP1s except PIP1;6 [38]. The anti-PIP2 antibody (R-2493) was raised against an amino peptide from ZmPIP2;4 and was shown to recognize all maize PIP2s (Dr. F. Chaumont personal communication). Following incubation in blocking solution (5% BSA in phosphate buffer solution) and in the anti-PIP1, PIP2, and preimmune antibodies (1:1000 dilution by volume), the sections were incubated in the fluorescein-coupled goat anti-rabbit IgG antibody (Sigma-Aldrich Canada Ltd., ON, Canada). The slides were examined under the fluorescence microscope (Leica DMRXA Upright Microscope) with a green light filter I3 at 450–490 nm excitation and 510 nm emission.

To determine the specificity of polyclonal antibodies, sodium dodecyl sulfate-polyacrylamide gel electrophoresis (12% acrylamide resolving gel) was performed with proteins (30 μg) extracted from the roots of a two-year old tamarack (*Larix laricina*) and two-week old greenhouse-grown maize (*Zea mays*). Following electrophoresis, gels were electroblotted to PVDF membrane, blocked overnight in Tris-buffered saline containing 5% (w/v) bovine serum albumin and probed with primary polyclonal antibodies (1:500) raised against *Zea mays* PIP1 and PIP2 aquaporins that were used for the immunolocalization [38]. Secondary antibody (1:10,000) raised in goat against rabbit IgG and conjugated to alkaline phosphatase (Sigma-Aldrich) was used to detect immunoreactive bands.

### Statistical analysis

The data were analyzed using SAS (version 9.1, SAS Institute Inc.; Cary, NC, USA). *T*-test was used to determine significant differences between treatment means of water relation and physiological parameters at α = 0.05. Analysis of variance (ANOVA) with the Mixed Procedure of SAS was used to determine differences in L_IND_ of individual roots at different temperatures at α = 0.05.

## Authors’ contributions

MC-P carried out and designed the experiments, performed statistical analyses of the data and drafted the manuscript. JS was in charge of growing plants and flooding treatments, assisted in measurements, and prepared tissue sections for microscopy. JJZ was responsible for designing the study, interpreting results co-drafting and editing the manuscript. All authors have read and approved the manuscript.

## Supplementary Material

Additional file 1 **SDS Page of the proteins extracted from maize (1) and tamarack (2) roots and stained with Coomassie blue (left).** Immunoblot of maize (1) and tamarack (2) proteins probed with ZmPIP1 antibody R-4445 (right). Click here for file
