# Supplementary figures and images for: Role of adventitious roots in water relations of tamarack (Larix laricina) seedlings exposed to flooding
Source: BMC Plant Biol. 2012 Jun 27;12:99. doi: 10.1186/1471-2229-12-99 (PMC3431261; doi:10.1186/1471-2229-12-99)

## Slide 1
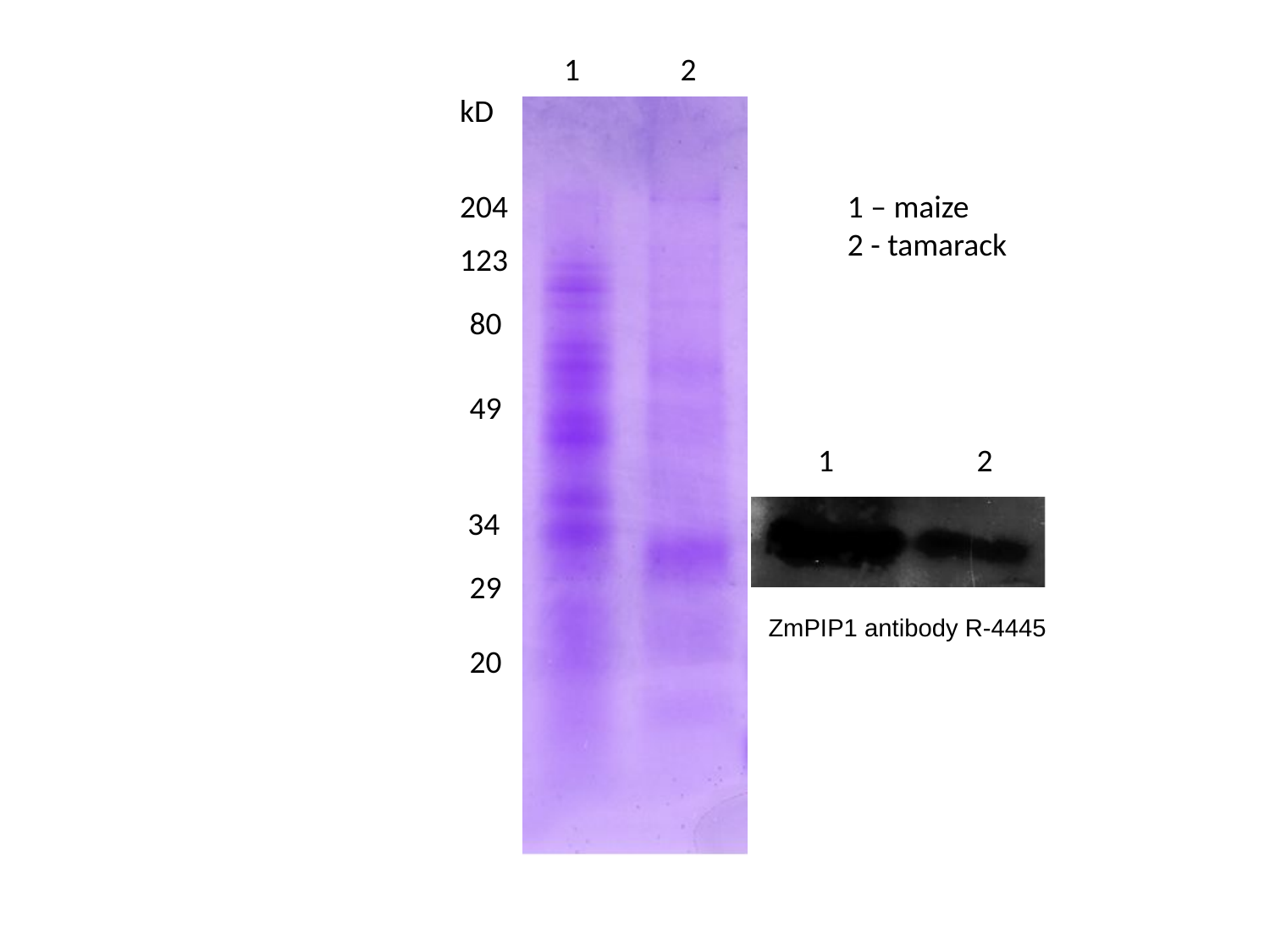

1
2
kD
204
1 – maize
2 - tamarack
123
80
49
1
2
34
29
ZmPIP1 antibody R-4445
20

Supplement: Additional file 1 — SDS Page of the proteins extracted from maize (1) and tamarack (2) roots and stained with Coomassie blue (left). Immunoblot of maize (1) and tamarack (2) proteins probed with ZmPIP1 antibody R-4445 (right). [file 1471-2229-12-99-S1.ppt]
